# Identifying the Superior Reperfusion Technique in Liver Transplantation: A Network Meta-Analysis

**DOI:** 10.1155/2019/9034263

**Published:** 2019-09-18

**Authors:** Yao Yao, Ping Wu, Tao Guo

**Affiliations:** ^1^School of Medicine, Huanggang Polytechnic College, Huanggang 438002, China; ^2^Department of Hepatobiliary and Pancreatic Surgery, Zhongnan Hospital of Wuhan University, Wuhan 430071, China

## Abstract

**Objective:**

To investigate the clinical effects of different reperfusion techniques in liver transplantation based on network meta-analysis.

**Method:**

Literature retrieval was conducted in globally recognized databases, namely, MEDLINE, EMBASE, and Cochrane Central, to address relative randomized controlled trials (RCTs) investigating the clinical effects of respective reperfusion techniques in liver transplantation. Short- and long-term parametric data, including ICU stay, dysfunction rate (DFR), biliary complications (BC), 1-year graft survival (GS), and patient survival (PS), were quantitatively pooled and estimated based on the Bayesian theorem. The *P* values of surface under the cumulative ranking (SUCRA) probabilities regarding each parameter were calculated and ranked by various techniques. The Grades of Recommendations Assessment, Development and Evaluation (GRADE) criteria were utilized for the recommendations of evidence from pairwise direct comparisons.

**Results:**

Seven RCTs containing 6 different techniques were finally included for network meta-analysis. The results indicated that retrograde vena cava (RVC) reperfusion possessed the highest possibility of revealing the best clinical effects on DFR (SUCRA, *P* = 0.93), ICU stay (SUCRA, *P* = 0.76), and GS (SUCRA, *P* = 0.44), while portal-arterial reperfusion (simultaneous initialize) seemed to exhibit the most benefits in reducing BC (SUCRA, *P* = 0.67) and enhancing PS rate (SUCRA, *P* = 0.48). Moreover, sensitivity analysis with the inconsistency approach clarified the reliability of the main results, and the evidence of the most direct comparisons was ranked low or very low.

**Conclusions:**

Current evidence demonstrated that RVC and portal-arterial reperfusion (simultaneously initialized) revealed superior clinical effects, compared to other interventions. Investigation of these 2 techniques should be a future research direction, and more high-quality RCTs are expected.

## 1. Introduction

Liver transplantation has witnessed advancements in surgical techniques in the last few decades, and the development of its peri- and intraoperative management has brought remarkable clinical efficacy [1, 2]. Nevertheless, normalization of some crucial intraoperative procedures was still needed, such as reperfusion techniques. It is known that the period of the greatest haemodynamic instability during liver transplantation occurs at graft reperfusion due to the insufficient preload for the vasodilatation of the splanchnic bed [3–5]. At the same time, ischaemia reperfusion damage is one of the main factors affecting graft function after liver transplantation, especially with regard to bile ducts that are highly susceptible to oxygen deprivation and reperfusion injury [6]. Thus, the whole revascularization process might induce the haemodynamic and metabolic disorders known as the postreperfusion syndrome, which can lead to postoperative failure and mortality [7]. To resolve this technical limitation, various reperfusion techniques have been applied to explore superior methods. Formerly, the most common technique of revascularization of the graft in liver transplantation was portal revascularization, followed by reconstruction of the hepatic artery inflow. The reason for this sequence is to ensure that the recipient liver receives blood in the shortest possible time, as portal vein anastomosis is easier, technically, than hepatic artery anastomosis [8, 9]. However, simultaneous arterial and portal anastomosis is currently feasible due to the improvement of haemodynamic management [10]. Moreover, portocaval shunts minimize portal congestion, although the duration of the anhepatic stage is longer, giving rise to more types of reperfusion techniques [11], and these improvements offered more space for ameliorating reperfusion techniques to avoid high postreperfusion syndrome rates.

Conversely, the debate over the merits and drawbacks of respective reperfusion techniques persists. There were 2 previous pair-wised meta-analysis that failed to resolve this debate due to a lack of systematic and comprehensive comparisons. Additionally, they failed to detail relative techniques and recommend any research directions for future clinical aims [12, 13]. Surprisingly, no comprehensive and quantitative network comparisons have yet been reported in this field to guide the next step in clinical improvement. Therefore, it is necessary to perform a network meta-analysis to determine the superior reperfusion technique in liver transplantation. More importantly, this study was undertaken to provide objective options for clinical decision-making and to discover new directions for clinical trials.

## 2. Methods

### 2.1. Literature Search and Retrieval

This study was conducted in strict accordance with the previously established PRISMA guidelines [14]. The retrieval for this study was initialized in global recognized electronic databases, including MEDLINE, EMBASE, and Cochrane Central, to avoid regional bias. MeSH terms individually or in combination were used to address relative trials that reported the comparisons of different reperfusion techniques in liver transplantation (details in Supplementary [Supplementary-material supplementary-material-1]). We did not apply any restrictions on publication status or publication date. However, full English texts had to be addressed if the trial was considered for inclusion.

### 2.2. Study Eligibility Criteria

The inclusion criteria of this study were based on the following: (1) randomized, controlled trials (RCTs); (2) studies comparing different reperfusion techniques in liver transplantation; and (3) studies providing available parameters of interests.

The following items were defined as the exclusion criteria: (1) non-RCTs; (2) no available parametric data reported; (3) studies focusing on basic science or other graft transplantation; (4) reviews, case reports, or comments; (5) repeated reports; (6) vague descriptions of reperfusion techniques; and (7) reperfusion techniques for cadaveric liver procurement.

### 2.3. Data Extraction and Outcomes of Interest

General information (e.g., author name, publication data, and region) and intervention-related characteristics (e.g., sample size and reported parameters) were abstracted using a predesigned form. To select outcomes of interests considering the available and comprehensiveness of parametric data, we chose ICU stay, dysfunction rate (DFR), and biliary complications (BC) as short-term postoperative parameters and 1-year graft survival (GS) and patient survival (PS) as long-term parameters. All of the parametric data regarding these 5 indices were extracted for pooled estimation to make comprehensive judgements about the clinical effects of respective reperfusion techniques.

### 2.4. Quality Assessment and Recommendation of Evidence

Since we only included RCTs for the current study, we applied the Cochrane Risk of Bias assessment tool [15] to evaluate the bias risk of individual studies with the following requirements: (1) free of selection bias; (2) free of performance bias; (3) free of detection bias; (4) free of attrition bias; (5) free of reporting bias; and (6) free of other biases. A graphic summary of the overall and study-level risk of bias was conducted using Review Manager Software (version 5.3). Furthermore, the Grades of Recommendations Assessment, Development and Evaluation (GRADE) criteria were utilized to assess the methodological quality of evidence in the current study [16]. Five factors (research limitations, inconsistent findings, uncertain direct evidence, inaccuracy or wide confidence intervals, and publication bias) to upgrade evidence and three factors (effect size, possible confounding factors, and dose-effect relationship) to downgrade evidence were detailed and reviewed for a final rating using GRADE profiler software (version 3.6). The process of quality assessment and GRADE rating were performed by group unanimous discussion.

### 2.5. Statistical Analysis

In the current study, we aimed to comprehensively evaluate the clinical effects of various reperfusion techniques based on the Bayesian theorem. It incorporates both direct and indirect information through a common comparator to obtain estimates of the relative interventional effects on multiple intervention comparisons [17, 18]. The *P* values of surface under the cumulative ranking (SUCRA) probabilities based on the consistency model are presented to clarify the pros and cons of different reperfusion techniques. The highest *P* value represented the possibility of exhibiting the best clinical effects according to respective parameters [19, 20]. Odds ratios (ORs) derived from network meta-analysis were calculated to exhibit the comparison of different techniques. Moreover, both a consistency model and an inconsistency model were conducted to test for the complete sensitivity and reliability of main results, while Potential Scale Reduction Factor (PSRF) values were limited to 1 to complete the calculation. The data model of network meta-analysis was calculated using Aggregate Data Drug Information System automated software (ADDIS, version 1.16).

## 3. Results

### 3.1. Study Characteristics and Quality Assessments

**Figure 1:**
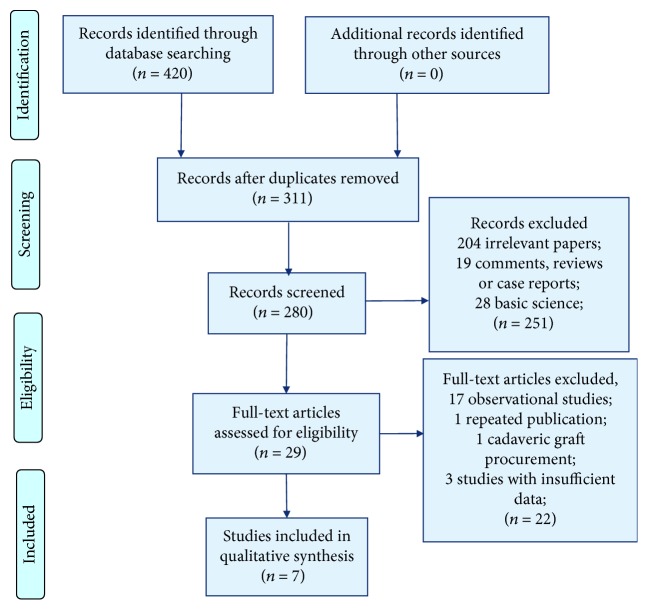
Flow diagram of the process of selecting studies for this network meta-analysis.

**Figure 2:**
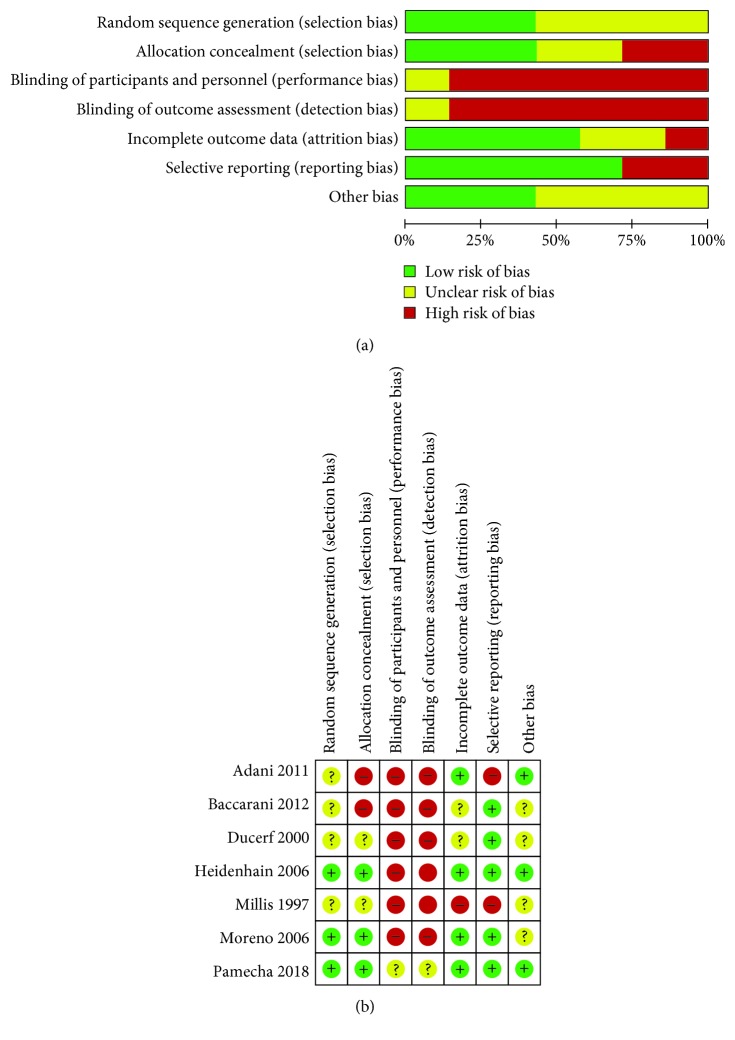
Bias assessment for included trials: (a) risk of bias graph presented as percentages across all of the included studies and (b) judgements regarding each risk of bias item for each included study.

**Figure 3:**
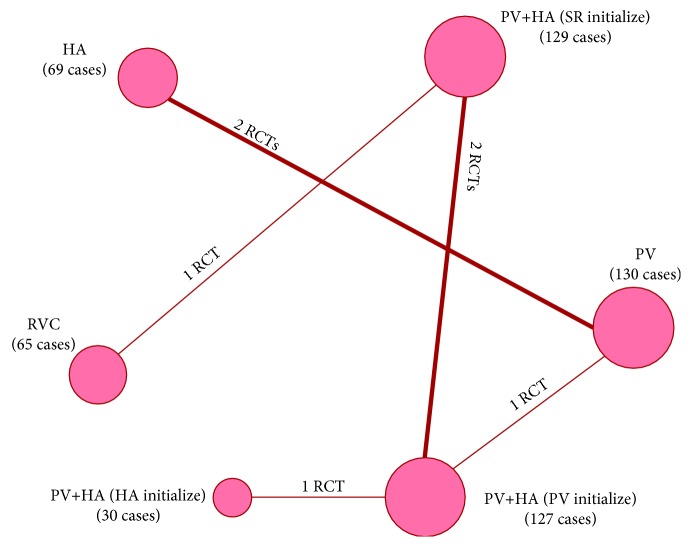
Network connections of all of the included trails. The numbers on the line indicate the quality of studies compared with every pair of procedures, which are also represented by the width of the lines. Additionally, the sizes of the areas of the circles indicate the respective sample sizes. PV: portal vein; HA: hepatic artery; RVC: retrograde vena cava; SR: simultaneous reperfusion.

After initially identifying 420 relative studies through systematic retrieval, we finally included 7 RCTs containing 550 patients for quantitative comparison [21–27] (Figure 1). All of them were basically performed by classic orthotopic liver transplantation or piggyback technique (Table 1). Most of these 7 RCTs were conducted without blinding approaches, yet selective reporting bias was not apparent either (details in Figure 2). Moreover, 4 reperfusion techniques, namely, retrograde vena cava (RVC) reperfusion, hepatic artery (HA) reperfusion, portal vein (PV) reperfusion, and HA plus PV reperfusion, were addressed. Based on these techniques, the HA plus PV reperfusion technique was divided into 3 different techniques according to the initialized vessels, which were PV initialize, HA initialize, and simultaneous reperfusion (SR) initialize. Thus, 6 different reperfusion techniques were classified for the final evaluations (details in Table 1 and Figure 3).

### 3.2. Results of the Network Meta-Analysis

**Figure 4:**
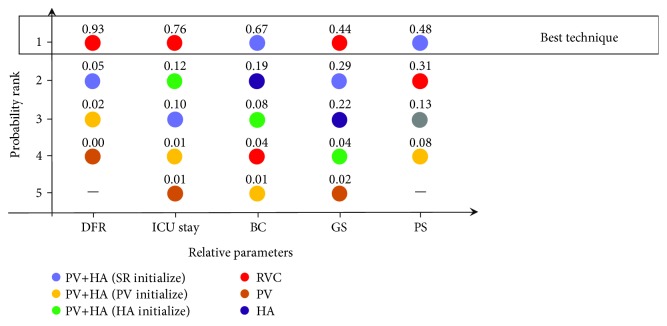
Plot of surface under the cumulative ranking curve values of respective techniques regarding different parameters. DFR: dysfunction rate; BC: biliary complications; GS: graft survival; PS: patient survival.

To assess short-term parametric data, we performed quantitative estimation based on DFR, ICU stay, and BC. DFR was used to describe the abnormal biochemical indices and graft injury. In the current study, DFR was calculated with the rate of postoperative primary/initial poor function, which were defined as significant hepatic enzymological aberration (including 1 presence or more of the following: bilirubin ≥ 10 mg/dl, INR > 1.6, and ALT or AST > 2000 U/ml) within postoperative 7 days [28, 29]. There were 4 included trails reporting relative raw data about DFR (Table 1). After a quantitative comparison, we discovered that RVC reperfusion seemed to be the best technique to achieve the lowest DFR (*P* = 0.93). To a certain extent, the length of postoperative ICU stay reflects the recovery time of liver function, and 5 trials provided relative parametric data. Based on the results of network meta-analysis, we illustrated that intraoperative RVC reperfusion could lead to the shortest ICU stay (*P* = 0.76). BC (biliary complications) were normally defined with imaging as strictures, dilatations, or other injuries of the bile ducts of the graft. Furthermore, it was used to reflect the safety of respective techniques in the current study. The objective results of pooled estimation regarding BC determined that PV plus HA (SR initialized) revealed superior clinical effects in reducing the postoperative BC rate, compared to other techniques (*P* = 0.67). In contrast, to evaluate the long-term effects of respective techniques, we conducted a comprehensive estimation regarding 1-year GS and PS. For GS, there were 5 trials reporting raw data for analysis, and the results of network meta-analysis indicated that RVC reperfusion techniques theoretically achieved the highest graft survival rate (*P* = 0.44), followed by the PV plus HA (SR initialize) technique (*P* = 0.29), while for the comparison of PS, we verified that the PV plus HA (SR initialize) reperfusion technique could bring the highest patient survival rate (*P* = 0.48), followed by the RVC technique (*P* = 0.31) (details presented in Figure 4 and Supplementary [Supplementary-material supplementary-material-1]). Therefore, according to these objective results, we found that intraoperative RVC and PV plus HA (SR initialize) reperfusion techniques seemed to reveal superior postoperative short- and long-term clinical effects.

### 3.3. Data Consistency and Quality of Evidence

To discover the steadiness and reliability of the main results in this study, we also performed quantitative analysis based on an inconsistency model. The results implicated that relative ORs and credible intervals were similar with the data of the consistency model (Supplementary [Supplementary-material supplementary-material-1]), while PSRF was always limited to 1 in each data operator. Thereby, we showed that our results were reliable, and no inconsistent risk existed. In addition, the GRADE rating demonstrated the quality of the evidence regarding respective parameters. All of the evidence was rated low or very low, indicating that the recommendation of evidence was limited (Supplementary [Supplementary-material supplementary-material-1]).

## 4. Discussion

In the current study, we included 7 RCTs containing 550 patients within 6 detailed reperfusion techniques to complete the quantitative network meta-analysis. For short-term effects, our results demonstrated that RVC reperfusion possessed the highest probability of reducing the postoperative dysfunction rate (SUCRA for DFR, *P* = 0.93) and ICU stay days (SUCRA for ICU stay, *P* = 0.76), yet PV plus HA (SR initialize) revealed the highest probability of achieving the lowest rate of postoperative biliary complications (SUCRA for BC, *P* = 0.67). At the same time, for long-term effects, we illustrated that RVC and PV plus HA (SR initialize) could result in the highest survival rate for grafts (SUCRA for BC, *P* = 0.44) and patients (SUCRA for BC, *P* = 0.48), respectively (Figure 4). Based on all of these objective results, we could conclude that RVC and PV plus HA (SR initialize) reperfusion seemed to be superior to other techniques, yet the conclusions still need to be further discussed.

Reperfusion is a crucial step in liver transplantation. Anatomically, PV or HA reperfusion alone can result in incomplete perfusion; thus, the most commonly used procedure for revascularization of the liver graft is initial reperfusion via the portal vein and subsequent reconstruction of the hepatic artery [30–32]. The aim of reperfusion is to reduce postreperfusion syndrome and ameliorate postoperative liver function. Consequently, the direction of improvement in reperfusion techniques is consistent in finding ways to achieve more complete perfusion in a shorter period of time. Improved surgical technique and haemodynamic management provide adequate time to complete synchronous revascularization of the PV and HA. Then, it was possible to conduct simultaneous reperfusion through the portal vein and hepatic artery [21, 22]. We know that delayed reconstructive arterial inflow in an exclusively portal reperfused graft potentially prolongs the warm ischaemia time to bring biliary strictures. Further, biliary stenosis was demonstrated to be associated with hepatic artery thrombosis or stenosis and with prolonged warm ischaemia time [33–35]. Therefore, compared to sequential portal-arterial reperfusion, simultaneous reperfusion can reveal some potential advantages; that is, the graft receives a more adequate blood supply during the critical phase of reperfusion within shorter arterial warm ischaemia to decrease damage to the biliary tract. Moreover, simultaneous reperfusion made it possible to conduct arterial anastomosis without retrograde bleeding from the graft hepatic artery in the surgical field. Conversely, conduction of simultaneous reperfusion can lead to a longer intraoperative anhepatic phase, which can result in a longer postoperative liver function recovery period. These reasons are why simultaneous reperfusion showed great effects in reducing biliary complications yet no superiority in decreasing the dysfunction rate and ICU stay. Conversely, another innovative technique, namely, retrograde vena cava reperfusion, has also received attention in clinical practice due to it subverting the traditional perfusion concept [35]. Basic experimental science demonstrated that oxygen free radicals play a crucial role during the early phase of reperfusion [36, 37]. Further, current studies focus on minimizing the cellular damage caused by oxygen free radicals. Theoretically, the oxygenized arterial blood of the hepatic artery contributes to generating more oxygen free radicals, both in sequential and in simultaneous portal-arterial reperfusions [38]. Therefore, it was hypothesized that low-pressure perfusion with low oxygenated blood could reduce the production of oxygen free radicals. For clinical practice, RVC reperfusion seems to result in a lower postreperfusion syndrome rate and has great benefits in reducing the dysfunction rate [12, 39]. However, the RVC technique was still debated and was not widely accepted due to it leading to longer ischaemic time and biliary damage. These facts were consistent with our result that RVC reperfusion could have better clinical effects on liver function recovery, thus decreasing DFR and shortening ICU stays, yet there seemed to be no benefit to avoiding postoperative biliary complications. Thus, in summary, based on our results and previous basic theories, both RVC and PV plus HA (SR initialized) reperfusion techniques seemed to reveal better clinical effects than other techniques. Even for long-term survival rates, RVC and PV plus HA (SR initialize) reperfusions also, respectively, possessed higher graft and patient survival rates than other techniques.

For the first time, we performed a comprehensive network meta-analysis to quantitatively compare respective reperfusion techniques in liver transplantation regarding different parametric data. Based on the current objective results and previous discussion, we could tentatively conclude that RVC and PV plus HA (SR initialize) reperfusion techniques were superior to other techniques, and we also admitted that both of these techniques have their own merits and disadvantages. Additionally, there was only one trial directly comparing these 2 procedures, so we could not determine which is superior for now; thereby, we raise this point to suggest new directions for clinical trials. Moreover, according to our previous discussion, we discovered that PV plus HA within SR initialization could lead to longer functional recovery time but lower biliary complication rates. Correspondingly, RVC reperfusion could result in faster graft recovery yet more postoperative biliary complication, and interestingly, both techniques revealed superior long-term effects. Therefore, we reckoned that the development of reperfusion techniques should focus on the reduction of reperfusion time, especially for HA, and the achievement of more anatomical perfusion at the same time. We thereby put forward another hypothesis whether RVC and PV plus HA could be synchronously performed in the future using constructive artificial vessels, for instance. This topic could be another field for future investigation.

Thus far, we have drawn preliminary conclusions and have raised future research directions through this study, yet some shortcomings existed. First, we only included 7 trials containing 550 cases for the analysis, and the inadequate trials and small sample size could contribute to the instability of our conclusions, although we demonstrated the consistency of our data. Meanwhile, the quality of evidence was not sufficient, which might also have potential impacts on our results. Second, our main results still needed some more validations. For instance, we quantitatively analyzed the DFR and PS as parts of the main results. However, each comparison only contained 4 trails, which may bring uncertainties to our main results. What is more, some important outcomes were not reported in current analysis. For example, vascular complication (such as hepatic artery or portal vein thrombosis) was clearly related to different reperfusion techniques and might have impacts on graft and patient's survival. But relative analysis could not be conducted due to inadequate raw data, and the quantitative analyzing was difficult to finish with an absent rate in some study arms. Third, the included trials were performed with nonuniform surgical techniques of hepatectomy and outflow reconstruction. And the details of procedures in some trails were not specifically elucidated, which made it difficult to conduct further subgroup and correlation analysis. Therefore, these confounding factors might also bring uncertain impacts on our results. Finally, according to our purposes and restrictive criteria, we might have omitted some high-quality literature. Therefore, we believe that more relatively high-quality RCTs with larger samples need to be conducted in the future.

In general, despite the existence of several limitations, we demonstrated the superior clinical effects of RVC and PV plus HA with SR-initialized reperfusion techniques regarding short- and long-term parametric data. More importantly, we provided options for clinical decision-making and raised new clinical research directions for the future.

## Figures and Tables

**Table 1:** Characteristics of included trails.

Author	Year	Region	Study arm	Sample size	Surgical technique	Interventional technique	Parameters
Adani et al.	2011	Italy	2	40	Piggyback technique	PV+HA (PV initialize) vs. PV+HA (SR initialize)	DFR; ICU stay; BC; GS; PS
Baccarani et al.	2012	Italy	2	80	Piggyback technique with outflow anastomosis done on three hepatic veins	PV+HA (PV initialize) vs. PV+HA (SR initialize)	DFR; ICU stay; BC; GS; PS
Ducerf et al.	2000	France	2	59	Piggyback technique with outflow anastomosis at the level of the left and median hepatic veins	PV vs. HA	BC
Heidenhain et al.	2006	Germany	2	131	Orthotopic liver transplantation with supra and infrahepatic end`-to-end cava anastomosis	PV+HA (SR initialize) vs. RVC	DFR; ICU stay; BC; GS; PS
Millis et al.	1997	USA	2	100	Orthotopic liver transplantation with venovenous bypass	PV vs. HA	BC; GS
Moreno et al.	2006	Spain	2	60	Piggyback technique	PV+HA (PV initialize) vs. PV+HA (HA initialize)	ICU stay; BC;
Pamecha et al.	2018	India	2	80	Piggyback technique with end-to-side single anastomosis of right hepatic vein and neo middle hepatic vein to the inferior vena cava	PV vs. PV+HA (PV initialize)	DFR; ICU stay; BC; GS; PS

PV: portal vein; HA: hepatic artery; RVC: retrograde vena cava; SR: simultaneous reperfusion; DFR: dysfunction rate; ICU: intensive care unit; BC: biliary complications; GS: graft survival; PS: patient survival.

## Supplementary Materials

Supplementary Materials Table S1: example search strategy and process in MEDLINE. Table S2: comparisons of included techniques in network meta-analysis regarding respective parameters. Odds ratios are presented in the cells in common between the column-defining and row-defining techniques. Table S3: sensitivity analysis—quantitative comparisons of included techniques based on inconsistency models. Table S4: quality of the evidence from direct comparisons regarding respective parameters.
