# Supplementary material for: Identifying the Superior Reperfusion Technique in Liver Transplantation: A Network Meta-Analysis
Source: Gastroenterol Res Pract. 2019 Sep 18;2019:9034263. doi: 10.1155/2019/9034263 (PMC6766671; doi:10.1155/2019/9034263)
Supplement: Supplementary Materials — Table S1: example search strategy and process in MEDLINE. Table S2: comparisons of included techniques in network meta-analysis regarding respective parameters. Odds ratios are presented in the cells in common between the column-defining and row-defining techniques. Table S3: sensitivity analysis—quantitative comparisons of included techniques based on inconsistency models. Table S4: quality of the evidence from direct comparisons regarding respective parameters. [file 9034263.f1.pdf]

Table S1. Example search strategy and process in MEDLINE.

# No.	Search	Results
1	exp (liver) OR hepatic/	1115728
2	exp (graft) OR allograft/	324001
3	1 AND 2	32719
4	exp transplantation/	704703
5	3 AND 4	29789
6	exp (flush) OR reperfusion/	99761
7	prospective	852082
8	random	331022
9	randomized controlled trial	601021
10	7 OR 8 OR 9	1618262
11	5 AND 6 AND 10	252

Table S2. Comparisons of included techniques in network meta-analysis regarding respective parameters. Odds ratios are presented in the cells in common between the column-defining and row-defining techniques.

Parameter	Comparison [OR (95%CI)]					
Regarding DFR	PV	0.18 (0.02, 1.47)	0.06 (0.00, 1.14)	0.01 (0.00, 0.49)		
	5.43 (0.68, 47.77)	PV + HA (PV initialize)	0.36 (0.06, 2.27)	0.08 (0.01, 1.26)		
	15.71 (0.87, 251.94)	2.81 (0.44, 16.86)	PV + HA (SR initialize)	0.23 (0.03, 1.75)		
	67.26 (2.02, 2082.22)	11.88 (0.79, 170.79)	4.37 (0.57, 33.36)	RVC		
Regarding ICU-stay	PV	-4.59 (-11.61, 2.19)	-3.92 (-9.11, 1.05)	-5.86 (-12.11, -0.06)	-7.63 (-15.34, -0.33)	
	4.59 (-2.19, 11.61)	PV + HA (HA initialize)	0.61 (-4.26, 5.36)	-1.30 (-7.29, 4.52)	-3.09 (-10.36, 4.32)	
	3.92 (-1.05, 9.11)	-0.61 (-5.36, 4.26)	PV + HA (PV initialize)	-1.94 (-5.32, 1.42)	-3.70 (-9.17, 2.10)	
	5.86 (0.06, 12.11)	1.30 (-4.52, 7.29)	1.94 (-1.42, 5.32)	PV + HA (SR initialize)	-1.76 (-6.17, 2.98)	
	7.63 (0.33, 15.34)	3.09 (-4.32, 10.36)	3.70 (-2.10, 9.17)	1.76 (-2.98, 6.17)	RVC	
Regarding BC	HA	1.80 (0.17, 21.74)	0.80 (0.01, 34.89)	0.60 (0.02, 15.41)	0.18 (0.00, 6.03)	1.02 (0.01, 58.44)
	0.56 (0.05, 5.93)	PV	0.48 (0.02, 8.10)	0.35 (0.02, 2.71)	0.10 (0.01, 1.24)	0.56 (0.02, 15.38)
	1.24 (0.03, 66.67)	2.10 (0.12, 52.36)	PV + HA (HA initialize)	0.62 (0.10, 5.69)	0.18 (0.02, 2.60)	0.96 (0.05, 36.97)
	1.68 (0.06, 53.53)	2.88 (0.37, 41.98)	1.60 (0.18, 9.96)	PV + HA (PV initialize)	0.31 (0.08, 1.12)	1.72 (0.14, 23.60)
	5.60 (0.17, 216.69)	9.81 (0.81, 181.37)	5.48 (0.38, 47.47)	3.24 (0.90, 12.72)	PV + HA (SR initialize)	5.90 (0.70, 54.93)
	0.98 (0.02, 69.66)	1.78 (0.07, 58.04)	1.04 (0.03, 20.45)	0.58 (0.04, 7.32)	0.17 (0.02, 1.42)	RVC
Regarding GS	HA	0.53 (0.09, 2.97)	0.76 (0.05, 12.27)	2.10 (0.08, 77.60)	2.46 (0.05, 130.53)	
	1.88 (0.34, 10.99)	PV	1.41 (0.17, 13.85)	3.90 (0.25, 85.74)	4.41 (0.16, 179.20)	
	1.32 (0.08, 21.93)	0.71 (0.07, 5.98)	PV + HA (PV initialize)	2.76 (0.45, 23.82)	3.16 (0.23, 56.19)	
	0.48 (0.01, 13.09)	0.26 (0.01, 3.95)	0.36 (0.04, 2.20)	PV + HA (SR initialize)	1.17 (0.19, 7.13)	
	0.41 (0.01, 18.91)	0.23 (0.01, 6.10)	0.32 (0.02, 4.28)	0.86 (0.14, 5.34)	RVC	
Regarding PS	PV	1.43 (0.25, 8.58)	2.67 (0.32, 26.23)	1.80 (0.11, 33.86)		
	0.70 (0.12, 4.01)	PV + HA (PV initialize)	1.85 (0.54, 7.27)	1.36 (0.15, 12.13)		
	0.38 (0.04, 3.15)	0.54 (0.14, 1.86)	PV + HA (SR initialize)	0.72 (0.10, 4.00)		
	0.56 (0.03, 9.40)	0.74 (0.08, 6.88)	1.39 (0.25, 9.85)	RVC		

Table S3. Sensitivity analysis: quantitative comparisons of included techniques based on inconsistency models.

Parameter	Comparison [OR (95%CI)]					
Regarding DFR	PV	0.18 (0.02, 1.42)	0.07 (0.00, 0.92)	0.01 (0.00, 0.44)		
	5.61 (0.71, 43.50)	PV + HA (PV initialize)	0.36 (0.05, 2.09)	0.09 (0.01, 1.25)		
	15.29 (1.09, 257.18)	2.77 (0.48, 19.48)	PV + HA (SR initialize)	0.23 (0.03, 1.84)		
	66.71 (2.28, 2295.39)	11.76 (0.80, 182.28)	4.26 (0.54, 33.52)	RVC		
Regarding ICU-stay	PV	-4.73 (-11.66, 2.05)	-4.14 (-9.21, 1.19)	-6.08 (-12.06, -0.03)	-7.76 (-15.30, -0.01)	
	4.73 (-2.05, 11.66)	PV + HA (HA initialize)	0.65 (-4.03, 5.43)	-1.36 (-6.86, 4.52)	-3.02 (-10.10, 4.41)	
	4.14 (-1.19, 9.21)	-0.65 (-5.43, 4.03)	PV + HA (PV initialize)	-2.05 (-5.34, 1.23)	-3.65 (-9.29, 2.18)	
	6.08 (0.03, 12.06)	1.36 (-4.52, 6.86)	2.05 (-1.23, 5.34)	PV + HA (SR initialize)	-1.64 (-6.32, 3.11)	
	7.76 (0.01, 15.30)	3.02 (-4.41, 10.10)	3.65 (-2.18, 9.29)	1.64 (-3.11, 6.32)	RVC	
Regarding BC	HA	1.51 (0.16, 24.02)	0.64 (0.01, 38.07)	0.48 (0.02, 15.70)	0.14 (0.00, 6.99)	0.76 (0.01, 70.66)
	0.66 (0.04, 6.38)	PV	0.43 (0.02, 8.31)	0.32 (0.03, 2.86)	0.10 (0.01, 1.26)	0.52 (0.02, 15.73)
	1.57 (0.03, 69.43)	2.33 (0.12, 64.04)	PV + HA (HA initialize)	0.76 (0.09, 5.87)	0.23 (0.02, 2.53)	1.25 (0.05, 37.75)
	2.10 (0.06, 51.97)	3.10 (0.35, 37.52)	1.31 (0.17, 10.56)	PV + HA (PV initialize)	0.31 (0.08, 1.16)	1.63 (0.13, 24.44)
	6.97 (0.14, 214.63)	10.48 (0.79, 165.04)	4.34 (0.40, 50.06)	3.27 (0.86, 12.92)	PV + HA (SR initialize)	5.34 (0.67, 58.77)
	1.32 (0.01, 67.31)	1.91 (0.06, 61.07)	0.80 (0.03, 21.08)	0.61 (0.04, 7.52)	0.19 (0.02, 1.49)	RVC
Regarding GS	HA	0.52 (0.10, 3.19)	0.75 (0.05, 12.35)	2.17 (0.08, 72.45)	2.47 (0.05, 151.31)	
	1.92 (0.31, 10.39)	PV	1.41 (0.16, 13.60)	4.16 (0.24, 86.47)	4.78 (0.16, 188.15)	
	1.33 (0.08, 19.85)	0.71 (0.07, 6.06)	PV + HA (PV initialize)	2.84 (0.41, 24.37)	3.23 (0.25, 57.36)	
	0.46 (0.01, 12.31)	0.24 (0.01, 4.18)	0.35 (0.04, 2.42)	PV + HA (SR initialize)	1.16 (0.19, 7.38)	
	0.40 (0.01, 18.47)	0.21 (0.01, 6.29)	0.31 (0.02, 3.98)	0.86 (0.14, 5.28)	RVC	
Regarding PS	PV	1.38 (0.24, 9.18)	2.70 (0.31, 28.04)	1.84 (0.11, 39.80)		
	0.72 (0.11, 4.13)	PV + HA (PV initialize)	1.90 (0.50, 7.20)	1.31 (0.14, 13.50)		
	0.37 (0.04, 3.19)	0.53 (0.14, 2.02)	PV + HA (SR initialize)	0.71 (0.11, 4.27)		
	0.54 (0.03, 8.79)	0.76 (0.07, 7.39)	1.41 (0.23, 9.47)	RVC		

Table S4. Quality of the evidence from direct comparisons regarding respective parameters.

Parameter	Direct comparisons		Quality of the evidence (GRADE)
Regarding DFR	PV	PV + HA (PV initialize)	⊕(very low)
	PV + HA (PV initialize)	PV + HA (SR initialize)	⊕ ⊕(low)
	PV + HA (SR initialize)	RVC	⊕(very low)
Regarding ICU-stay	PV + HA (PV initialize)	PV	⊕(very low)
		PV + HA (HA initialize)	⊕(very low)
		PV + HA (SR initialize)	⊕ ⊕(low)
	PV + HA (SR initialize)	RVC	⊕(very low)
Regarding BC	PV	HA	⊕(very low)
	PV + HA (PV initialize)	PV	⊕(very low)
		PV + HA (HA initialize)	⊕(very low)
		PV + HA (SR initialize)	⊕ ⊕(low)
	PV + HA (SR initialize)	RVC	⊕(very low)
Regarding GS	PV	HA	⊕(very low)
	PV + HA (PV initialize)	PV	⊕(very low)
		PV + HA (SR initialize)	⊕ ⊕(low)
	PV + HA (SR initialize)	RVC	⊕(very low)
Regarding PS	PV + HA (PV initialize)	PV	⊕(very low)
		PV + HA (SR initialize)	⊕ ⊕(low)
	PV + HA (SR initialize)	RVC	⊕(very low)
